# Editorial: Global excellence in ethnopharmacology: europe

**DOI:** 10.3389/fphar.2024.1368610

**Published:** 2024-01-30

**Authors:** Andreana N. Assimopoulou, Adriana Trifan

**Affiliations:** ^1^ School of Chemical Engineering, Faculty of Engineering, Aristotle University of Thessaloniki, Thessaloniki, Greece; ^2^ Natural Products Research Centre of Excellence (NatPro-AUTh), Center for Interdisciplinary Research and Innovation, Aristotle University of Thessaloniki, Thessaloniki, Greece; ^3^ Department of Pharmacognosy-Phytotherapy, Faculty of Pharmacy, Grigore T. Popa University of Medicine and Pharmacy, Iași, Romania

**Keywords:** ethnopharmacology, traditional medicine, natural products, drug discovery, bioactive compounds

## Introduction

“*Global Excellence in Ethnopharmacology: Europe*” Research Topic is a special edition of the Frontiers in Pharmacology, that highlights the latest advancements in Ethnopharmacology in Europe, showcasing the academic excellence and high-quality work of internationally recognized researchers.

Ethnopharmacology is defined as “the interdisciplinary scientific exploration of biologically active agents traditionally employed or observed by man” (Bruhn and Holmstedt, 1981). We would define Ethnopharmacology as a key to deciphering the ancient medicinal codes. Most modern medications can be traced back to their origins in traditional remedies. Historically, natural products (NPs) have played a key role in drug discovery and served as a valuable source for novel molecular scaffolds in drug development, with around 65% of all approved drugs classified as NPs or inspired by a NP core (Davidson and Brimble, 2019; Newman and Cragg, 2020).

It is essential to obtain information on the bioactive compounds from well-documented useful plants, mostly medicinal ones, such as structure elucidation and therapeutic dose, their relative contribution to the effects of the extract (including synergistic or antagonistic effects), the toxicological profile of the extract and its constituents, alongside safety, molecular mechanism of action, etc. (modified by Heinrich et al., 2018a). Indeed, ethnopharmacologists focus on documenting the knowledge related to the local and traditional medicines, thus contributing to better healthcare at a community level. In addition, their studies should comply with the national and international laws and agreements (the Convention on Biological Diversity and subsequent treaties and regulations including the Nagoya Protocol), in order to promote sustainable harvesting and cultivation practices (access to local medicinal plants), to ensure the availability of these resources for future generations, and most importantly, to warrant benefit sharing with local communities (Heinrich et al., 2018b).

Traditional remedies discovered through ethnopharmacological research can serve as sources of new leads and inspiration for developing new drugs. The contribution of ethnopharmacology is widely acknowledged and credited and can further leverage to integrating traditional practice into modern healthcare systems and for the next-generation of therapeutics.

## Ethnopharmacology in Europe

This Research Topic aims to shed light on the recent progress made across the entire breadth of the Ethnopharmacology field in Europe and reflects on the future challenges faced by researchers across borders. This Research Topic includes manuscripts that focus on the following: i. Ethnopharmacological studies; ii. Phytochemical analysis of plants used in ethnopharmacology by advanced techniques; iii. Isolation and purification of bioactive compounds; iv. *In vitro* and *in vivo* findings on plants and plant extracts used in ethnopharmacology, as well as bioactive constituents, related to anti-inflammatory, diuretic, antimicrobial and antiviral activities and elucidation of their mechanism of action.

*Capparis cartilaginea* Decne. (CC) originates from the dry regions of Asia and the Mediterranean basin. In traditional medicine, tea of CC leaves was used to treat inflammatory conditions such as rheumatism, arthritis, and gout, but limited studies reported the phytochemistry and biological activity of CC. Alsharif et al. identified the chemical composition of CC extract using high-speed countercurrent chromatography (HSCC), Nuclear Magnetic Resonance (NMR) and High-Performance Liquid Chromatography coupled to Electrospray Ionisation and Quadrupole Time-of-Flight Mass Spectrometry (HPLC-ESI-QTOF-MS/MS). CC extract and tea were rich in phenolic compounds and exhibited a strong antioxidant activity. The *in silico* model applied presented excellent predictions of the relative inhibitory effect of the purified flavonoids on MMP-9, as their biological activity was consistent with the compounds’ binding affinity. CC extract and tea exhibited a significant anti-inflammatory effect, which can partially explain their traditional medicinal use.

Alkannin, shikonin and their derivatives (A/S) are secondary metabolites produced in the roots of certain plants of the Boraginaceae family, such as *Lithospermum erythrorhizon* Siebold & Zucc. and *Alkanna tinctoria* (L.) Tausch. These naphthoquinones exert anti-cancer, wound healing, and antimicrobial activities and are approved active pharmaceutical ingredients of strong wound healing pharmaceutical preparations, invented through ancient scripts by Prof. Papageorgiou. In the work of Rat et al., the interactions between endophytic bacteria isolated from *A. tinctoria* and the antimicrobials A/S were studied. Endophytic bacteria known to be resistant to the compounds were screened for their effect on A/S in liquid medium. The strain *Pseudomonas* sp. R-72008 was selected. Bacterial growth was recorded, and high-performance liquid chromatography-diode array and ultra-high performance liquid chromatography-electrospray ionization mass spectrometry analyses were performed to detect and quantify metabolites. In nutrient medium inoculated with R-72008, a decrease in the amount of A/S monomers initially present was observed and correlated with an increase of A/S oligomers. Moreover, a significant decrease of initial A/S monomers in minimal medium was correlated with bacterial growth, showing for the first time that a bacterial strain, *Pseudomonas* sp. R-72008, was able to use the naphthoquinones A/S as sole carbon source. This study opens new perspectives on the interactions between bacteria and plant antimicrobials.

Tsioutsiou et al. reported an ethnopharmacological study of medicinal plants used against skin ailments on Mount Pelion, Central Greece, a study area not been investigated up to date. The information on the medicinal uses of the various species was obtained through extensive semi-structured interviews or the completion of questionnaires by the informants. The elaboration of the gathered information included the calculation of some quantitative indices, such as Fidelity Level (FL), and Informant Consensus Factor (FIC). Moreover, the relative importance of each reported species was identified by calculating the Use Value (UV). The interviews revealed 38 plant taxa belonging to 27 plant families reported to be used in the study area exclusively against skin diseases. The plant family mostly mentioned by the informants was Hypericaceae, followed by Plantaginaceae and Amaryllidaceae, while among the most popular methods of application are cataplasms, compresses, and topical application of decoction or raw plant material. Some of the most cited species are *Hypericum perforatum* L., *Quercus coccifera* L., and *Plantago* spp., traditionally used to treat skin problems such as eczema, wounds, and insect stings. The present ethnopharmacological study is the first documentation of ethnobotanical knowledge of this area that points out the traditional uses of medicinal plants against skin ailments.

Emanuel et al. assessed the *in vivo* effects of *Pelargonium sidoides* DC. root extract EPs^®^ 7,630 in SARS-CoV-2-infected hamsters and investigated the properties of EPs^®^ 7,630 and its functionally relevant constituents in phenotypically distinct SARS-CoV-2 variants. EPs^®^ 7,630 reduced viral load early in the course of infection and displayed significant immunomodulatory properties positively modulating disease progression in hamsters. EPs^®^ 7,630 differentially inhibited SARS-CoV-2 variants in nasal and bronchial human airway epithelial cells. Antiviral effects were more pronounced against Omicron BA.2 compared to B.1 and Delta, the latter two preferring TMPRSS2-mediated fusion with the plasma membrane for cell entry instead of receptor-mediated low pH-dependent endocytosis. By using SARS-CoV-2 Spike VSV-based pseudo particles (VSVpp), the authors confirmed higher EPs^®^ 7,630 activity against Omicron Spike-VSVpp, which seems independent of the serine protease TMPRSS2, suggesting that EPs^®^ 7,630 targets endosomal entry. They identified at least two molecular constituents of EPs^®^ 7,630, i.e., (−)-epigallocatechin and taxifolin with antiviral effects on SARS-CoV-2 replication and cell entry.

*Thymus comosus* Heuff ex. Griseb. (Lamiaceae) is a wild thyme species endemic for Romanian Carpathian areas, frequently collected as substitute for collective herbal product Serpylli herba, cited as antibacterial and diuretic remedy in traditional medicine. The study of Babotă et al. evaluated the *in vivo* diuretic effect using Wistar rats treated orally with each herbal preparation and the *in vitro* antimicrobial properties of three herbal preparations (infusion—TCI, tincture—TCT and an hydroethanolic extract prepared through an optimized ultrasound-assisted method—OpTC) obtained from the aerial parts of T. comosus. All the extracts exerted a mild diuretic action, TCT and OpTC inducing the most intense diuretic effect. Both herbal preparations produced a statistically significant, dose-dependent and gradual increase of the urine output. Potentiometric evaluation of urine samples collected from treated rats revealed a clear and mild natriuretic and kaliuretic effect after the administration. In terms of antimicrobial activity, *E. coli*, *B. cereus*, *Penicillium funiculosum* and *P. verrucosum* var. *cyclopium*, showed the greater sensitivity to the tested extracts, respectively. UHPLC-HRMS screening showed that the bioactive potential of *T. comosus* herbal preparations was likely related to the higher amounts of phenolic acids, flavonoids and other phenolics. The obtained results support the ethnopharmacological evidence regarding the mild diuretic and antibacterial potentials of the endemic wild thyme *T. comosus*, this study being the first one that assessed the afore-mentioned bioactivities for this species.

In summary, ethnopharmacology serves as a vital link and bridge between traditional wisdom, ancient scripts and contemporary scientific exploration. It plays a pivotal role in substantiating and harnessing the therapeutic strategies from diverse cultures, simultaneously enriching our comprehension of natural remedies and their putative advantages. Ethnopharmacology has already re-emerged as an innovative approach for modern drug discovery and there are a lot of expectations, given the advancements of scientific fields that can contribute in the new era (computational approaches, molecular networking, molecular pharmacology, -omics technologies, natural products chemistry and technology, artificial intelligence).

This Research Topic has comprehensively addressed significant research in ethnopharmacology and traditional medicines in Europe. These studies can provide support for clinical practice and the interpretation of mechanisms of traditional medicine and may further inspire novel concepts and adequate future research.
